# Up-regulation of ST18 in pemphigus vulgaris drives a self-amplifying p53-dependent pathomechanism resulting in decreased desmoglein 3 expression

**DOI:** 10.1038/s41598-022-09951-x

**Published:** 2022-04-08

**Authors:** Sari Assaf, Dan Vodo, Kiril Malovitski, Janan Mohamad, Shir Bergson, Yarden Feller, Liron Malki, Ofer Sarig, Eli Sprecher

**Affiliations:** 1 Division of Dermatology, Tel Aviv Sourasky Medical Center, 6, Weizmann street, 64239 Tel Aviv, Israel; 2 Department of Human Molecular Genetics and Biochemistry, Sackler Faculty of Medicine, Tel Aviv University, Tel Aviv, Israel

**Keywords:** Translational research, Genetics research, Skin diseases, Autoimmunity

## Abstract

Pemphigus vulgaris (PV) is a life-threatening autoimmune mucocutaneous blistering disease which is to a large extent genetically determined, and results, at least in part, from the deleterious activity of autoantibodies directed against desmoglein (DSG)3, a prominent intra-epidermal adhesion molecule. Those autoantibodies lead to decreased membranal DSG3 expression in keratinocytes (KCs), thereby destabilizing cell–cell adhesion within the epidermis and leading to blister formation. We previously showed that rs17315309, a strong risk variant for PV within the promoter of the *ST18* transcription factor gene, promotes epidermal ST18 up-regulation in a p53/p63-dependent manner. Accordingly, ST18 was found to be overexpressed in the skin of PV patients. Increased ST18 expression was then shown to markedly augment PV autoantibodies-mediated loss of KCs cohesion. Here, we demonstrate that ST18 overexpression significantly increases autoantibody-mediated DSG3 down-regulation in keratinocytes. In addition, DSG3 decreased expression boosts p53 function through p38 mitogen-activated protein kinase (p38MAPK) activation and dramatically augments p53-dependent *ST18* promoter activity. Finally, the PV risk variant rs17315309 is associated with increased p53 expression in PV skin. Taken collectively, these observations reveal a novel self-amplifying pathomechanism involving ST18, DSG3, p38 and p53, capable of perpetuating disease activity, and therefore indicative of novel actionable molecular targets in PV.

## Introduction

PV is an autoimmune condition most commonly diagnosed during the fifth to seventh decade of life^1^. The disease manifests with flaccid blisters over the skin and mucosal surfaces, leading to painful and non-healing erosions that without appropriate treatment can result in life-threatening infections and metabolic disturbances^2^.

PV annual incidence is estimated at 0.76–50 new cases per million with significant variability across ethnic groups^3^. Thanks to the advent of corticosteroids and more advanced systemic immunosuppressive treatments, mortality rates in PV have dropped to below 5%, with therapies-related adverse effects accounting for most PV-associated deaths^4^.

PV is caused by autoantibodies directed against desmosomal proteins (such as DSG3 and DSG1) as well as other autoantigens, leading to loss of adhesion (acantholysis) between KCs and consequently to intraepidermal blistering^5–7^. Additional mechanisms have also been implicated in PV IgG-induced blister formation^8^.

The propensity to develop PV is to a large extent, genetically determined^9,10^. A significant number of risk variants have been identified at the HLA locus^11^. In addition, a number of non-HLA genes and loci have been found to be associated with PV in various populations^9,10^. Among these, association studies^12–14^ have revealed in some but not all populations, several PV-associated risk variants in the vicinity of the *ST18* gene which encodes a transcription factor involved in skin inflammation and apoptosis^15^. Deep sequencing of the *ST18* gene locus revealed a functionally relevant risk variant (rs17315309) within the gene promoter which was shown to up-regulate *ST18* promoter activity^16^. Accordingly, ST18 expression was found to be increased in the skin of PV patients^14,17^. ST18 increased expression was in turn found to boost tumor necrosis factor (TNFα) expression by KCs and to enhance PV IgG-induced KCs acantholysis^16–18^. Of note, the rs17315309 risk variant was found to promote ST18 expression in a p53-dependent manner^16^. Further underscoring the possible role of p53 in PV pathogenesis, depletion of DSG3 in KCs was shown to result in increased p53 expression and activity^19,20^.

Here we demonstrate that ST18 overexpression steers a p53-dependent process resulting in enhanced autoantibody-mediated membranal DSG3 down-regulation in KCs, revealing a novel and pivotal self-perpetuating pathomechanism in PV.

## Results

### ST18 overexpression increases antibody-mediated DSG3 down-regulation

**Figure 1:**
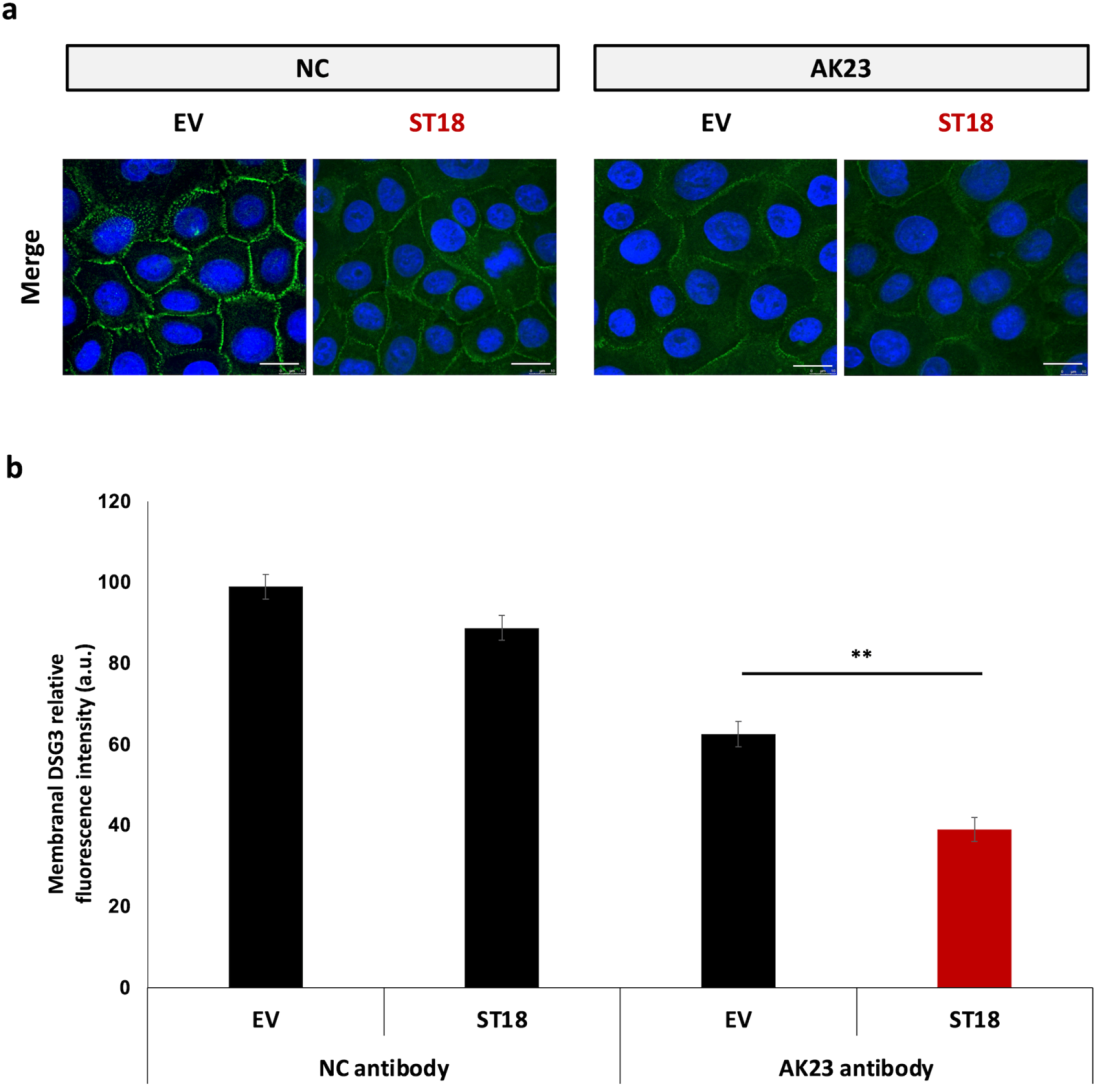
ST18 enhances antibody-mediated DSG3 down-regulation in keratinocytes. (**a**) NHEKs were transfected with an ST18 expression vector (ST18) or with a control empty vector (EV); 24 h post transfection, cells were exposed to AK23 for 12 h and were then fixed and immunostained for DSG3 (green signal) and DAPI (blue signal); (**b**) Expression of DSG3 was quantified by ImageJ software. Results represent the mean ± SE of three independent experiments (**p < 0.01 by 2-tailed t test; scale bar = 20um).

Given the importance of DSG3 for normal cell–cell adhesion in the epidermis and its central role in the pathogenesis of PV^6,8^, we initially assessed the effect of ST18 up-regulation on antibody-mediated DSG3 down-regulation in keratinocytes. Normal human keratinocytes (NHEKs) were transfected with an ST18 expression vector or an empty vector as a control and were then exposed to AK23, a pathogenic monoclonal antibody targeting the DSG3 N-terminus which induces loss of epidermal cell–cell adhesion^21,22^. Using immunofluorescent (IF) staining (Fig. 1a), ST18 overexpression was shown to significantly enhance AK23-mediated down-regulation of membranal DSG3, as compared to NHEKs transfected with the empty vector (Fig. 1b).

We then compared DSG3 membrane expression between AK23-exposed cells displaying ST18 expression and AK23-exposed cells lacking ST18 expression. As seen in Supplementary Fig. 1, cells overexpressing ST18 exhibited significantly increased AK23-mediated DSG3 down-regulation, as compared to neighbouring cells negative for ST18 overexpression.

### DSG3 down-regulation results in enhanced expression and activity of p53

**Figure 2:**
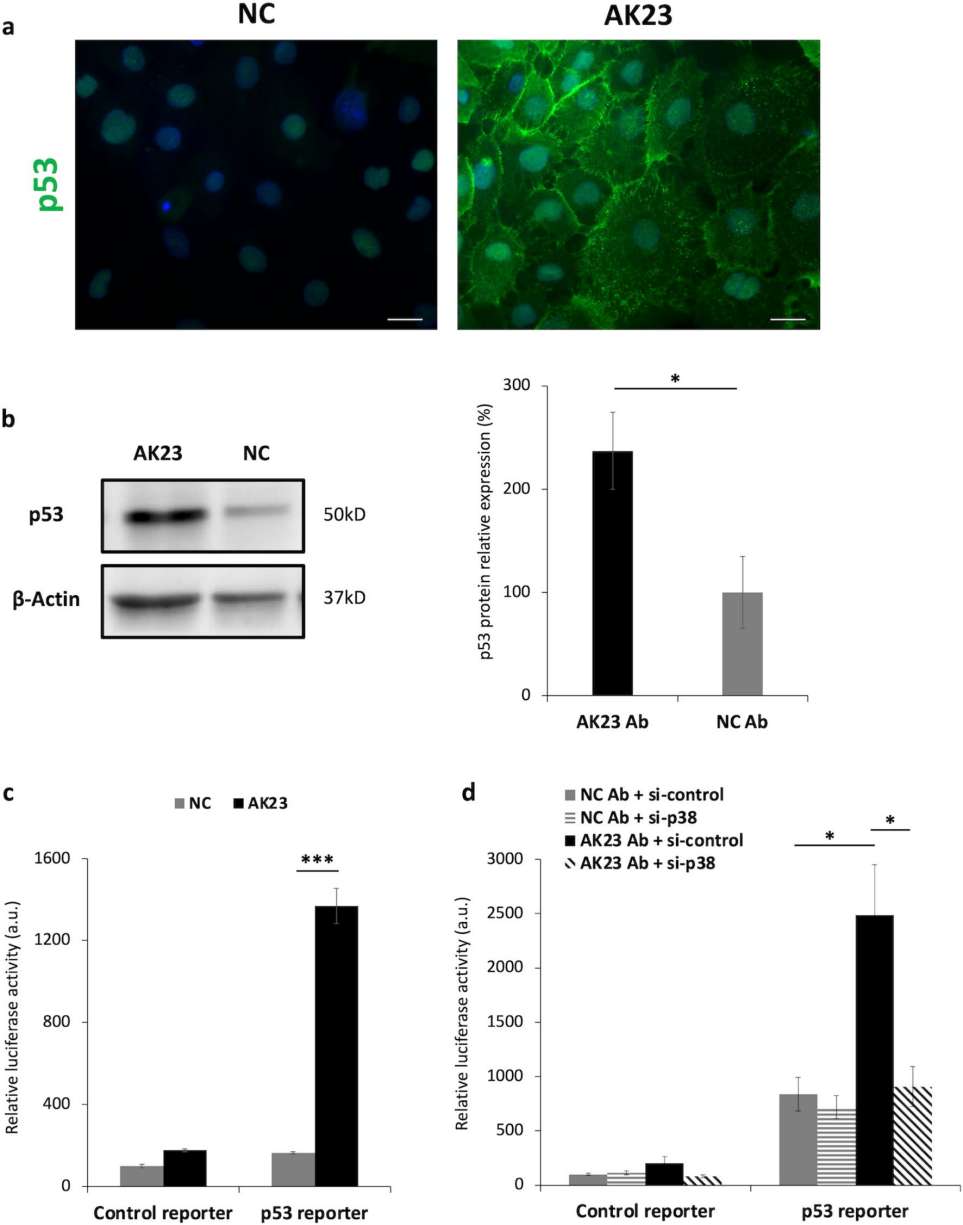
Antibody-mediated DSG3 down-regulation affects p53 expression and activity in a p38MAPK-dependent manner. (**a**) NHEKs exposed to either AK23 or negative control antibody (NC) were stained for p53 (green) and DAPI (blue) (scale bar = 20um); (**b**) p53 protein expression in NHEKs exposed to either AK23 or negative control antibody (NC) was assessed using immunoblotting with anti-p53 antibody. β-actin served as a loading control (left panel). Protein levels were quantified and data was normalized to levels observed in negative control antibody-treated cells (right panel). Results represent the mean ± SE of four independent experiments (*p < 0.05 by 2-tailed t test); (**c**) NHEKs were transfected with a luciferase reporter construct under the regulation of a p53 binding site or with a control reporter, and were then treated either with AK23 antibody or negative control antibody (NC). Results represent the mean ± SE of three independent experiments (***p < 0.001 by 2-tailed t test); (**d**) NHEKs were transfected with a luciferase reporter construct under the regulation of a p53 binding site or a control reporter. Cells were additionally transfected with control (si-control) or *p38MAPK*-specific (si-p38) siRNAs. Twenty four hours post-transfection, cells were treated either with AK23 antibody (AK23 Ab) or negative control antibody (NC Ab). Results represent the mean ± SE of four independent experiments (*p < 0.05 by one way ANOVA test). Original blots are presented in Supplementary Fig. 4.

Given the fact that ST18 up-regulation increases anti-DSG3 antibodies-mediated DSG3 down-regulation and given the fact that ST18 expression is under p53 regulation^16^, we next investigated the effect of autoantibody-mediated DSG3 down-regulation on p53 expression. A previous study showed that DSG3 knockdown was associated with increased expression and stabilization of p53^19^. NHEKs were cultured in the presence of AK23 or a negative control antibody and p53 expression was assessed using IF staining as well as Western blotting. As seen in Fig. 2a, NHEKs exposed to AK23 exhibited increased expression of p53 as compared to cells exposed to a non-pathogenic control antibody. Western blotting confirmed that antibody-mediated DSG3 down-regulation results in increased p53 expression (Fig. 2b). Similar results were obtained with NHEKs cultured in the presence of sera obtained from PV patients versus healthy controls (Supplementary Fig. 2).

We then ascertained the impact of DSG3 downregulation on p53 activity using a luciferase reporter under the regulation of a p53 binding motif. As seen in Fig. 2c, NHEKs exposed to AK23 exhibited significantly increased p53 transcriptional activity as compared to cells exposed to control antibody. Furthermore, as p38MAPK is known to up-regulate p53 activity^23,24^ and inhibition of p38MAPK blocks PV autoantibody-induced loss of intercellular adhesion^25^, we examined whether the effect of DSG3 downregulation on p53 activity is mediated through p38MAPK. As seen in Fig. 2d, upon silencing of p38MAPK using specific siRNA, AK23-induced activation of p53 in NHEKs was significantly decreased as compared to cells transfected with control siRNA, reaching levels similar to those measured in cells exposed to non-pathogenic antibodies. Similar results were observed upon DSG3 silencing using specific siRNA as compared to control siRNA (Supplementary Fig. 3).

### DSG3 down-regulation increases ST18 promoter activity in a p53-dependent manner

**Figure 3:**
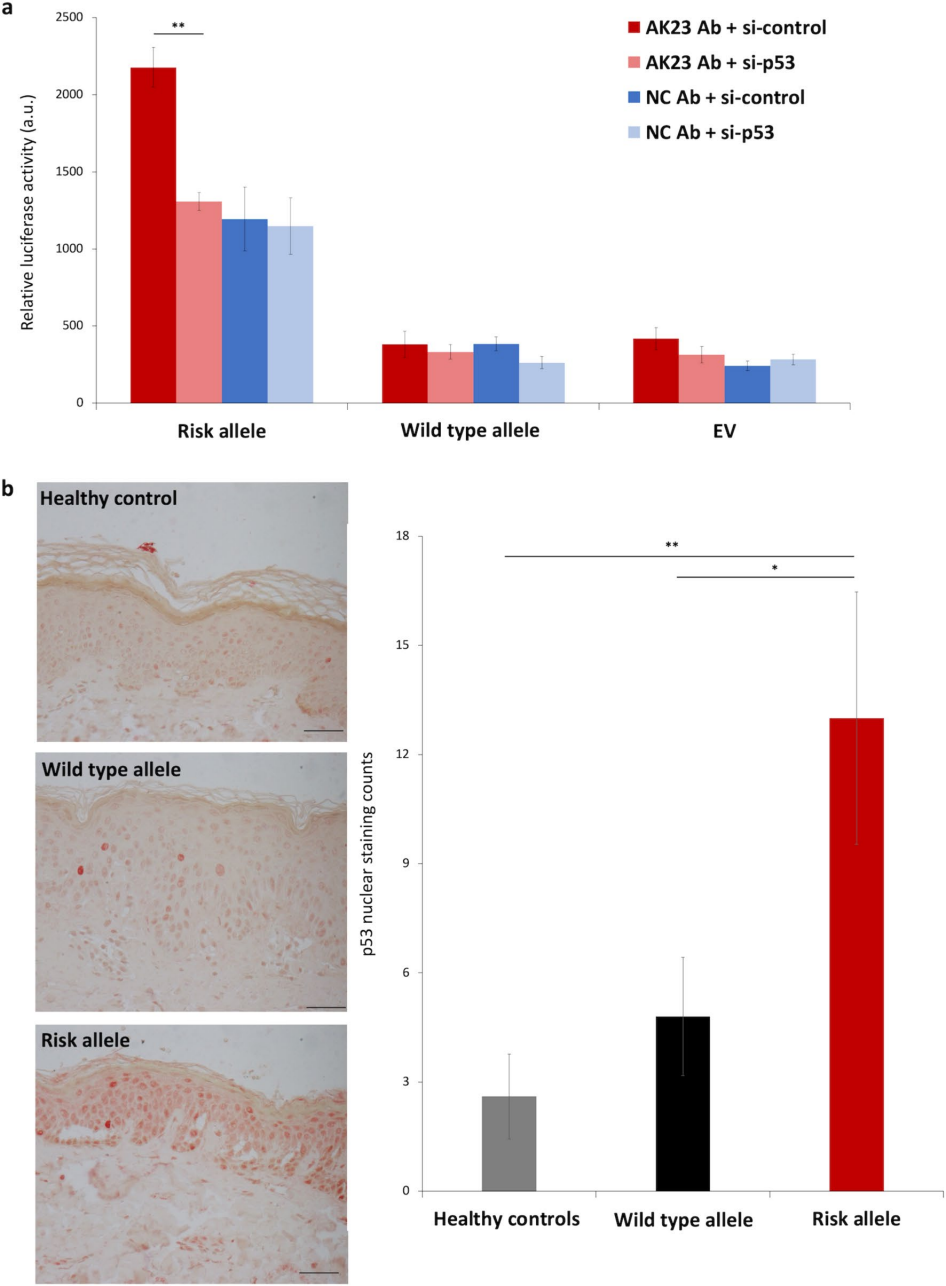
DSG3 down-regulation affects p53-dependent ST18 promoter activity. (**a**) NHEKs were transfected with a an empty vector (EV) as a control or with a luciferase reporter construct under the regulation of a 282 bp *ST18* promoter fragment harbouring either the rs17315309 wild type or risk alleles. Cells were additionally transfected with control (si-control) or *TP53*-specific siRNAs (si-p53). Twenty four hours post-transfection, cells were treated either with AK23 antibody (AK23 Ab) or negative control antibody (NC Ab). Results represent the mean ± SE of three independent experiments (**p < 0.01 by 2-tailed t test); (**b**) Immunohistochemistry (left panel) of p53 nuclear stain in skin biopsy samples obtained from PV patients carrying rs17315309 wild type (n = 5) or risk alleles (n = 3) or from healthy controls (n = 5) (scale bar = 100um). p53 nuclear staining was quantified (right panel) by two independent evaluators (*p < 0.05, **p < 0.01 by 2-tailed t test).

Given the facts that (1) p53 expression increases following DSG3 down-regulation (Fig. 2) and (2) p53 regulates ST18 expression^16^, we hypothesized that down-regulation of DSG3 may result in a p53-dependent increase in ST18 expression, which in turn may explain the association between ST18 expression in the epidermis and increased risk for PV^14^. To ascertain this possibility, NHEKs were transfected with a reporter construct consisting of the luciferase-encoding gene under the regulation of a 282 bp fragment derived from the *ST18* promoter region. This specific fragment spans a p53 binding motif that contains either the wild type (T) or the risk (C) allele of the PV-associated rs17315309 variant^16^. NHEKs were additionally transfected with *TP53*-specific or control siRNAs and were then cultured in the presence of AK23 or a control antibody. As seen in Fig. 3a, transfected cells grown in the presence of AK23 as compared to the control antibody displayed increased *ST18* promoter activity exclusively in cells expressing the construct harbouring the rs17315309 (C) risk allele. This effect was significantly decreased upon p53 silencing, thus demonstrating that DSG3 down-regulation promotes ST18 activity in a p53-dependent manner only in cells expressing the PV-associated risk allele.

### rs17315309, is associated with increased p53 expression

The rs17315309 (C) risk allele confers an heightened risk to develop PV^16^ as well as an increased risk for PV patients to develop an extensive disease^17^. To ascertain the clinical relevance of the above findings, we attempted at correlating carrier status for the rs17315309 (C) risk allele and p53 nuclear expression. Patients carrying the rs17315309 risk allele showed a significantly increased expression of nuclear p53, as compared to patients carrying the rs17315309 (T) wildtype allele or as compared to healthy individuals (Fig. 3b).

## Discussion

Broadening our understanding of PV pathogenesis, and more specifically of the role of non-immunological elements in blister formation, is expected to be instrumental for the development of alternative, effective and safer treatment strategies^6,8,26^. At this regard, a growing number of genetic and functional studies suggest a role for the transcription factor ST18 in PV pathogenesis^12,14,16–18^, adding to the existing body of evidence pointing to epidermal elements as playing a critical and potentially actionable role in PV pathogenesis^9,10^.

We have now shown that the propensity to develop PV is determined in several populations in part by a functional risk variant leading to ST18 up-regulation^14,16^. ST18 up-regulation is associated with KCs secretion of pro-inflammatory cytokines, including TNFα^16,17^, as well as aggravation of anti-DSG3-mediated destabilization of epidermal cell–cell adhesion^16^. We now show that ST18 induces DSG3 down-regulation, which in turn activates p53 which is required for ST18 up-regulation. Of interest, previous studies demonstrated that DSG3 binds to and inhibits p38MAPK^27^ which is capable of up-regulating p53^23,24^. Accordingly, we demonstrate here that activation of p53 by DSG3 down-regulation is dependent on p38MAPK activity. This in turn may explain the fact that inhibition of p38MAPK has been shown to prevent acantholysis in in vitro, ex vivo and in vivo models^28–30^. p38MAPK may also contribute to PV pathogenesis through inhibition of desmosome assembly via p38MAPK-mediated inactivation of the GTPase RhoA, which is involved in actin cytoskeleton reorganisation^31^. Interestingly, p53 was shown to inhibit RhoA activity^32^ while RhoA deficiency resulted in p53 activation^33^. Whether ST18 is also involved in this p38MAPK-dependent pathway remains to be investigated. In fact, the contribution of ST18 overexpression to PV pathogenesis is expected to be pleiotropic given that it functions as a transcription factor affecting the expression of multiple targets. For example, we recently demonstrated that ST18 also perturbs cell–cell adhesion through stimulation of TNF-α secretion^17^.

ST18 clearly affects keratinocyte biological activities. It is unclear whether it also affects the production of auto-antibodies to DSG3. In fact, we previously failed to demonstrate a correlation between the presence of a risk variant in *ST18* and anti-DSG3 antibody titers in PV patients^16^.

**Figure 4:**
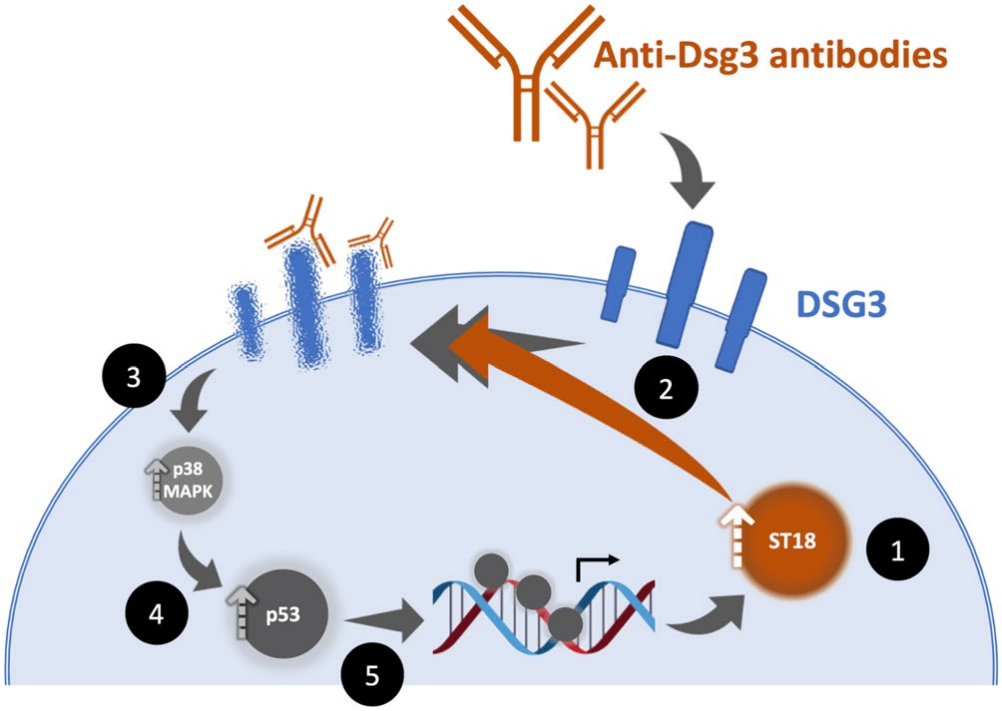
ST18 drives a p53-dependent self-amplifying process promoting autoantibody-mediated membranal DSG3 down-regulation in pemphigus vulgaris. Genetically determined ST18 overexpression (1) enhances autoantibodies-induced DSG3 down-regulation in keratinocytes (2, orange arrow) which triggers p38MAPK-dependent (3) p53 activity (4), which in turn up-regulates *ST18* promoter activity (5), thus setting the stage for a self-amplifying pathogenetic cycle in PV.

Taken collectively, the present and previous^14,16–18^ observations suggest that ST18 overexpression in PV skin, which is genetically determined^14,16^, boosts pro-inflammatory cytokine secretion and autoantibody-mediated DSG3 down-regulation, which result in impaired cell–cell adhesion within the epidermis^14,16,17^. The fact that DSG3 down-regulation stimulates p53 expression in a p38MAPK-dependent fashion sets the stage for a self-perpetuating mechanism which explains PV chronicity (Fig. 4). Thus, ST18 plays a pivotal role in autoimmunity propagation in PV, a fact which is reminiscent of other autoimmune disease processes^34^ and opens new avenues of research for treatment development and individualization.

## Methods

### Patients

All participants provided written informed consent in accordance with a protocol approved by the Tel Aviv Medical Center Institutional review Board and with the principles of the Declaration of Helsinki. PV and negative control serum samples collection was conducted as previously described^16,17^.

### Cell cultures

NHEKs were obtained from foreskin as previously described^16^. The cells were grown in Medium 154CF (Thermo Fisher, M154CF500) supplemented with Human Keratinocyte Growth Supplement Kit (HKGS Kit, Thermo Fisher, S1001K) in 6-well or 12-well plates. Prior to exposure to AK23, Ca^2+^ concentration was raised to 1.2 mM. For expression studies, NHEKs grown to ~ 70% confluence on 12-well plates were transfected with various constructs using lipofectamine 2000 (Life Technologies, Carlsbad, CA), as described elsewhere^16^ (Supplementary Fig. 4).

### Antibodies

NHEKs were exposed to monoclonal antibody AK23 (3.75 μg/ml) (Biozol, Eching, Germany, D219-3) or monoclonal mouse IgG1 antibody (3.75 μg/ml) (R&D Systems, Minneapolis, MN, MAB002). For IF and Western blotting studies, we used mouse monoclonal anti-p53 antibody (diluted 1:50; Santa Cruz Biotechnology, Santa Cruz, CA, DO-1), mouse monoclonal anti-desmoglein-3 antibody (5H10) conjugated to Alexa Fluor 488 (Novus Biologicals, LLC, NBP1-78984AF488), rabbit polyclonal anti-DDDDK tag (Binds to FLAG tag sequence) antibody (diluted 1:100, Abcam, Cambridge, UK, ab1162), secondary horseradish peroxidase-conjugated goat anti-mouse antibody (diluted 1:10,000; Jackson ImmunoResearch Laboratories, West Grove, PA, 115-035-003), mouse monoclonal anti β-actin antibody (Sigma-Aldrich, diluted 1:10,000, Abcam, Cambridge, UK, ab8224), mouse monoclonal anti α-tubulin antibody (diluted 1:5000, Sigma-Aldrich, T9026), secondary horseradish peroxidase-conjugated anti-rabbit antibody (diluted 1:5000; Sigma-Aldrich, 12-348) and rabbit polyclonal anti p38 MAPK antibody (diluted 1:1000 Cell Signaling Technology, Danvers, MA, USA, #9212).

### Constructs design and generation

A 282 bp *ST18* gene fragment spanning rs17315309 was PCR-amplified from DNA extracted from two patients homozygous for the rs17315309 wild-type T and minor C alleles. The resulting amplicons were cloned into pGL4.17 vector (Promega, Madison, WI, USA) upstream to the luciferase encoding gene, as described previously^16^. An 8.0 kb-fragment harboring the *ST18* open reading frame cloned into a FLAG-containing pCMV6-Entry vector (4.9 kb) was purchased from Origene Technologies Company (Rockville, MD, USA). The empty pCMV6-Entry was used as a control^16^.

### IF studies

NHEKs were grown on glass coverslips and fixed with 4% paraformaldehyde. Following permeabilization with 0.1% Triton/PBS, sections were blocked in 5% BSA and incubated overnight at 4 °C with primary antibodies. Secondary antibody staining was carried out for 1 h at 37 °C using goat anti-mouse IgG (H + L) cross-adsorbed secondary antibody, Alexa Fluor 488 (diluted 1:200, Invitrogen, Carlsbad, CA, USA, A-11001) or goat anti-rabbit IgG (H + L) cross-adsorbed secondary antibody, Rhodamine Red-X (diluted 1:500, Invitrogen, Carlsbad, CA, USA, #R-6394). Negative control staining without the primary antibodies is shown in Supplementary Fig. 5. Coverslips were mounted in polyvinyl alcohol. Imaging was done with Leica TCS SP5 confocal microscope (Leica Microsystems, Wetzlar, Germany). Image analysis was performed with ImageJ software (National Institutes of Health, Bethesda, MD).

### Western blotting

Cells were homogenized in CelLytic MT (Sigma-Aldrich) and a protease inhibitor mix, including 1 mM phenylmethanesulfonyl fluoride, and 1 mg/ml aprotinin and leupeptin (Sigma-Aldrich). Following centrifugation, at 10,000*g* for 10 min at 4 °C, proteins were electrophoresed through a 12.5% SDS-PAGE and transferred onto an Immun-Blot PVDF membrane (Bio-Rad, Hercules, CA, 1620177). After blocking for 1 h using 1 × Tris-buffered saline with Tween-20 (50 mM Tris, 150 mM NaCl, 0.01% Tween 20) with 5% BSA, blots were incubated overnight at 4 °C with mouse monoclonal anti-p53 antibody (diluted 1:200; Santa Cruz Biotechnology, Santa Cruz, CA, DO-1). The blots were washed five times for 5 min each with 1 × Tris-buffered saline, 0.1% Tween 20 with 1.5% BSA. After incubation with horseradish peroxidase-conjugated goat anti-mouse antibody (diluted 1:10,000; Jackson ImmunoResearch Laboratories, West Grove, PA), and subsequent washings (five times, 5 min each with 1 × Tris-buffered saline Tween-20), proteins were detected using the EZ-ECL chemiluminescence detection kit (Biological Industries, Beit Haemek, Israel). To compare the amount of protein in different samples, we reprobed the blots using a mouse monoclonal antibody to β-actin (Sigma-Aldrich). Protein levels were quantified by ImageJ software (National Institutes of Health, Bethesda, MD). Original Western blot membranes are provided in Supplementary Fig. 6.

### Immunohistochemistry studies

After antigen retrieval with 0.01 mol/L citrate buffer (pH 6.0; Invitrogen, Carlsbad, CA) in a microwave for 25 min, blocking with hydrogen peroxide for 10 min, and protein blocking for 40 min, 5-mm-thick paraffin-embedded sections fixed on Plus glass slides (Menzel Glaser, Braunschweig, Germany) were processed by using an automated immunostainer (Benchmark-XT; Ventana Medical System, Tucson, AZ) with mouse monoclonal anti-p53 antibody (diluted 1:20; Santa Cruz Biotechnology, Santa Cruz, CA, DO-1). Negative controls consisted of slides processed while omitting the primary antibody. Visualization of the bound primary antibodies was performed with the mouse and rabbit specific HRP/AEC (ABC) Detection IHC Kit (Abcam, Cambridge, UK, ab93705). Sections were then counterstained with Gill hematoxylin, dehydrated, and mounted for microscopic examination. Specimens were examined with a Nikon 50I microscope connected to a DS-RI1 digital camera (Nikon, Tokyo, Japan). Immunohistochemistry nuclear staining was quantified with ImageJ software (National Institutes of Health, Bethesda, Md) by two independent evaluators.

### p53 luciferase reporter assay

Primary keratinocytes (25,000 cells/well) cultured in a white flat-bottom 96-well microplate were transfected in the presence of Lipofectamine 2000 (Invitrogen, Grand Island, NY) with a luciferase reporter construct containing an artificial p53-responsive promoter (pRGC-luc) carrying 17 tandem repeats of the p53 consensus DNA binding sequence derived from the ribosomal gene cluster1 as previously described^35^ or an empty luciferase pGL2 as a negative control as well as a Renilla expression vector. Twenty four hours post transfection, Ca^2+^ concentration was increased to 1.2 mM and cells were treated with the monoclonal AK23 antibody (3.75 μg/ml) (Biozol, Eching, Germany, D219-3) or a monoclonal mouse IgG1 antibody (3.75 μg/ml) (R&D Systems, Minneapolis, MN,MAB002) as negative control. Twenty four hours later, a dual luciferase assay (Promega, Madison, Wisconsin) was used to measure luciferase activity, which was normalized to Renilla luciferase activity, using Tecan Infinite M200 device (Tecan Group Ltd, Männedorf, Switzerland). For p38MAPK silencing the same experiment described above was repeated. Briefly, primary keratinocytes were co-transfected with the same pGL2 vectors, Renilla expression vector and control siRNA (Life Technologies, Carlsbad, CA, 452002) or a specific p38 alpha MAPK14-specific siRNA (Santa Cruz Biotechnology, Santa Cruz, CA, SC-29433). Efficiency of gene knock down was assessed by qRT-PCR and Western blotting (Supplementary Fig. 7). For DSG3 silencing the experiment described above was repeated with additional co-transfection of the cells with a DSG3-specific siRNA (Santa Cruz Biotechnology, Santa Cruz, CA, SC-43115). Efficiency of gene knock down was assessed by qRT-PCR (Supplementary Fig. 3).

### ST18 promoter luciferase reporter assay

NHEKs (25,000 cells/well) cultured in white flat-bottom 96-well microplate were co-transfected in the presence of Lipofectamine 2000 (Invitrogen, Grand Island, NY) with a luciferase reporter harbouring an *ST18* gene promoter fragment spanning the rs17315309 wild-type (T) or risk (C) allele^16^, or with an empty luciferase pGL4.17 as a negative control and a Renilla expression vector. In some experiments, cells were concomitantly transfected with *TP53*-specific siRNA (Santa Cruz Biotechnology, Santa Cruz, CA, SC-29435) or control siRNA (Life Technologies, Carlsbad, CA, 452002). Efficiency of gene knock down was assessed by qRT-PCR and Western blotting (Supplementary Fig. 8). Twenty four hours post transfection, Ca^2+^ concentration was raised to 1.2 mM and cells were treated with the AK23 monoclonal antibody (3.75 μg/ml) (Biozol, Eching, Germany, D219-3) or a monoclonal mouse IgG1 antibody (3.75 μg/ml) (R&D Systems, Minneapolis, MN,MAB002). Fourty eight hours post transfection, a dual luciferase assay (Promega, Madison, Wisconsin) was used to measure luciferase activity, which was normalized to Renilla luciferase activity, using Tecan Infinite M200 device (Tecan Group Ltd, Männedorf, Switzerland).

### qRT-PCR and expression analysis

RNA was extracted from cultured cells using an RNA extraction kit (Roche, Mannheim, Germany). cDNA was synthesized and PCR amplified as previously done^36^. Cycling conditions were as follows: 95 °C, 20 s, followed by 95 °C, 3 s; 60 °C, 30 s for 40 cycles. Each sample was analyzed in triplicates. Results were normalized to *GAPDH* mRNA levels. Primers used for qPCR experiments are as follows: For *GAPDH* we used the primers 5’- GAGTCAACGGATTTGGTCGT -3’ and 5’- GACAAGCTTCCCGTTCTCAGCC -3’; for *p38MAPK* we used the primers 5’- GGTTACGTGTGGCAGTGAAGAAG -3’ and 5’- GCAGGTGTAAAAACGTCCAACAG -3’; for *TP53* we used the primers 5’- GAGCTGAATGAGGCCTTGGA -3’ and 5’-CTGAGTCAGGCCCTTCTGTCTT -3’; for *ST18* we used the primers 5’- AAAACTCACGGGAAGACAGAG -3’ and 5’- GGTTTAGGGCTTGGTATAGAGG -3’; for *DSG3* we used the primers 5’- GAGATGACTATGCAACAAGCT -3’ and 5’-TTCTCTACATCTAGTCCTTGG -3’. Each sample was analyzed in triplicates. Results were normalized to *GAPDH* mRNA levels.

### Statistical analysis

Comparisons of values between two groups were performed by the unpaired or paired Student’s *t*-test. When more than two groups were evaluated, one-way ANOVA was performed. A value of P < 0.05 was considered statistically significant.

## Supplementary Information

Supplementary Information.

## Data Availability

All data that support the findings of this study are available from the corresponding author upon request.
