# Supplementary material for: Up-regulation of ST18 in pemphigus vulgaris drives a self-amplifying p53-dependent pathomechanism resulting in decreased desmoglein 3 expression
Source: Sci Rep. 2022 Apr 8;12:5958. doi: 10.1038/s41598-022-09951-x (PMC8993920; doi:10.1038/s41598-022-09951-x)
Supplement: Supplementary file 1 — Supplementary Information. [file 41598_2022_9951_MOESM1_ESM.pdf]

Supplementary data

Up-regulation of ST18 in pemphigus vulgaris drives a self-amplifying p53-dependent pathomechanism resulting in decreased desmoglein3 expression

Sari Assaf[1,2,^], Dan Vodo[1,^], Kiril Malovitski[1,2], Janan Mohamad[1,2], Shir Bergson[1,2], Yarden Feller[1,2], Liron Malki[1,2], Ofer Sarig[1], Eli Sprecher[1,2]

[1]Division of Dermatology, Tel Aviv Sourasky Medical Center, Tel Aviv, Israel.

[2]Department of Human Molecular Genetics and Biochemistry, Sackler Faculty of Medicine, Tel Aviv University, Tel Aviv, Israel

^ Equal contributors

Supplementary figures

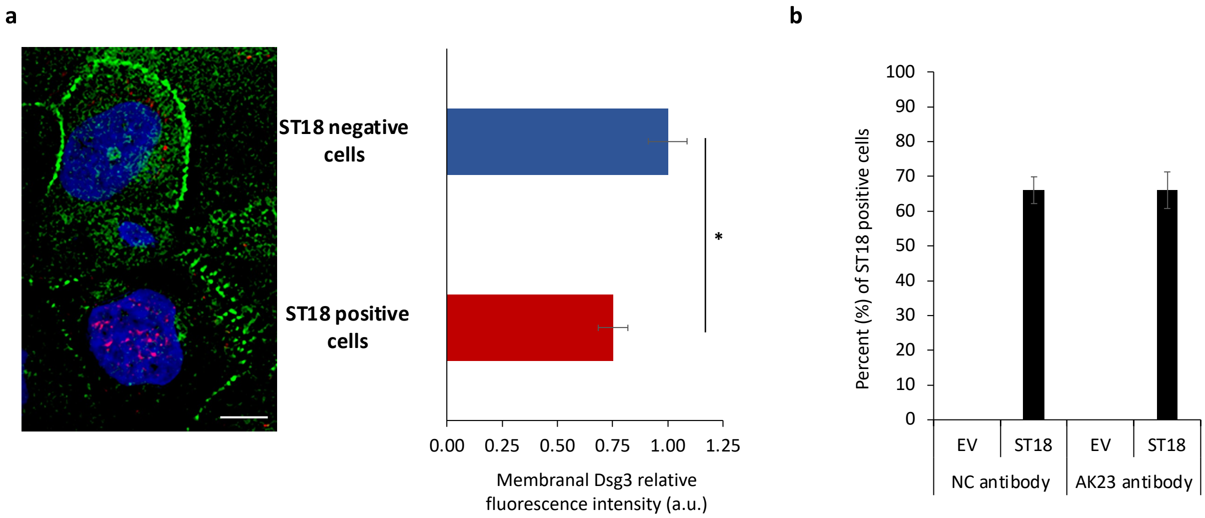

Supplementary Figure 1. ST18 overexpression increased AK23-induced DSG3 down-regulation.

(a) Normal human epidermal keratinocytes (NHEKs) were transfected with an ST18 expression vector. 24 hours post transfection, cells were exposed to AK23 for 12 hours and were then fixed and immunostained for DSG3 (green signal), Flag-tagged ST18 construct (red signal) and DAPI (blue signal). Expression of DSG3 was quantified by ImageJ software. Results represent the mean $\pm$ SE of three independent experiments (*$p<0.05$ by 2-tailed t test, scale bar=10um); (b) Cells positive for ST18 overexpression were counted in 10 different fields. Results represent the mean $\pm$ SE of three independent experiments.

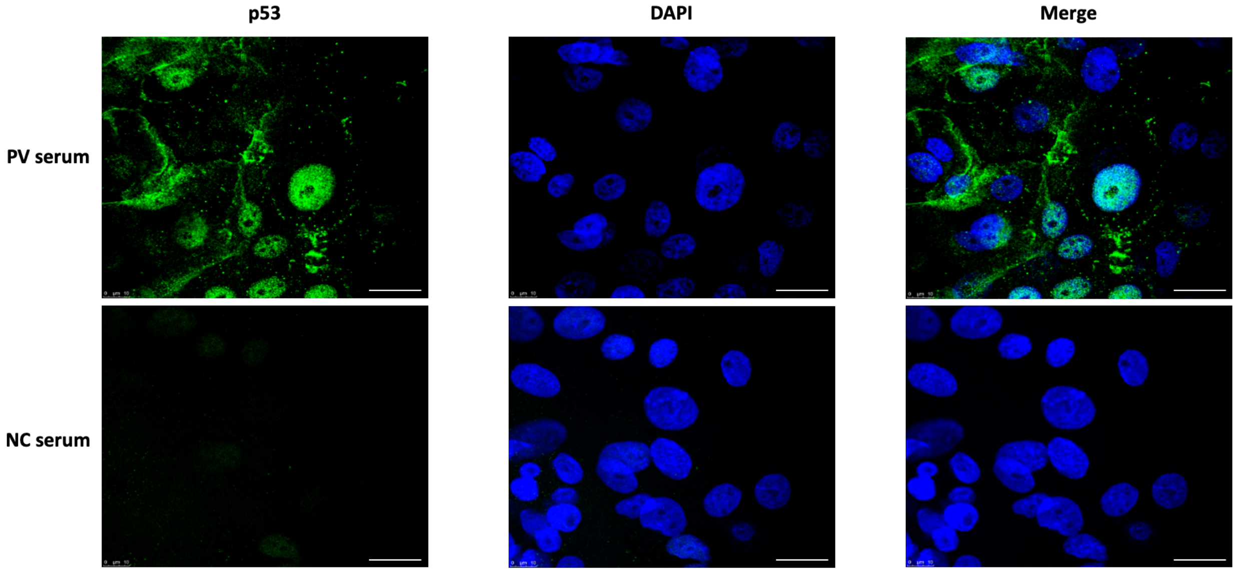

Supplementary Figure 2. PV serum-induced DSG3 down-regulation results in enhanced p53 expression.

NHEKs exposed to PV serum (PV serum, upper panel) or control serum (NC serum, lower panel) were stained for p53 (left column) and DAPI (middle column). Merged staining is shown in the right column (scale bar=20um).

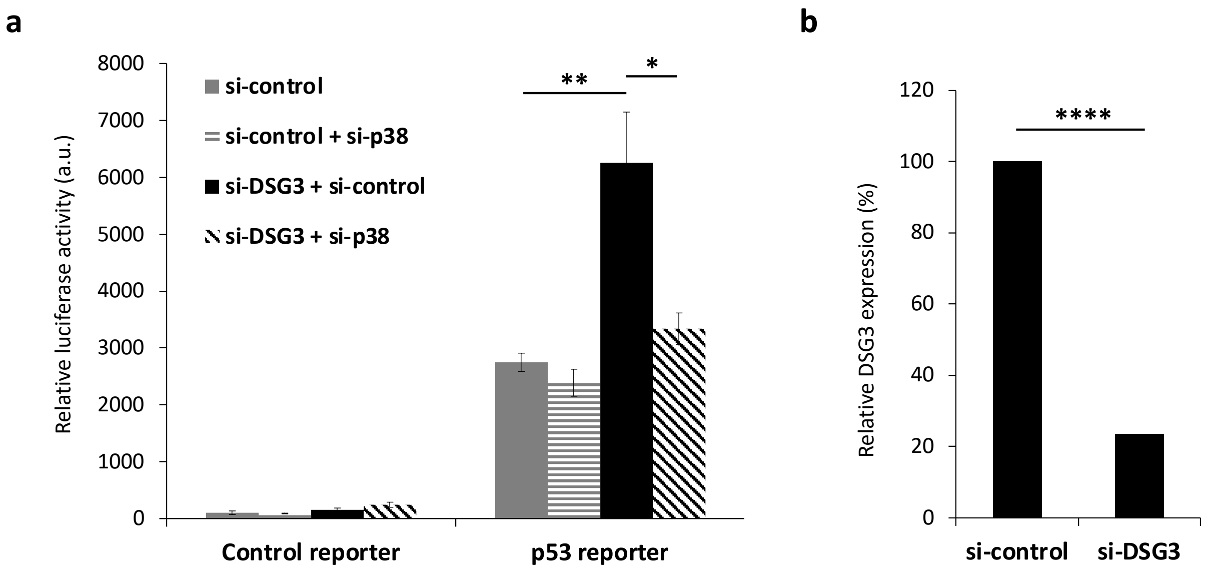

Supplementary Figure 3. DSG3 silencing affects p53 expression and activity in a p38MAPK-dependent manner

(a) NHEKs were transfected with a luciferase reporter construct under the regulation of a p53 binding site or a control reporter. Cells were additionally transfected with control (si-control) or *p38MAPK*-specific (si-p38) siRNAs as well as with *DSG3*-specific (si-DSG3) siRNAs. Results represent the mean ± SE of three independent experiments (*p<0.05, **p<0.01 by one way ANOVA test); (b) RNA levels of *DSG3* were quantified by qRT-PCR 24 hours post transfection. Results represent the mean ± SE of three independent experiments (****p<0.0001 by 2-tailed t test)

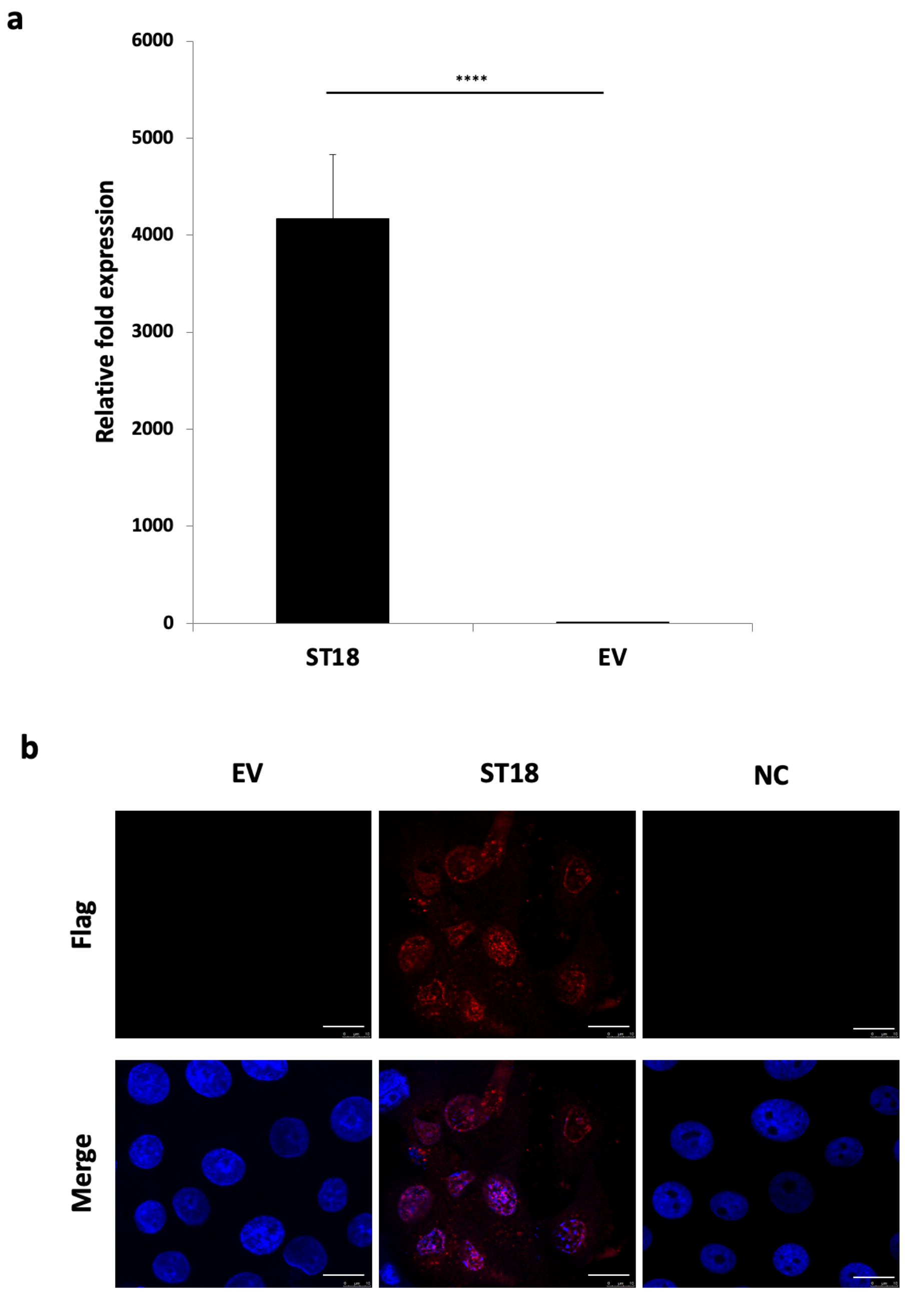

Supplementary Figure 4. ST18 overexpression validation.

NHEKs were transfected as described in Materials and Methods with an ST18-expressing vector (ST18) or an empty vector (EV). (a) RNA levels were quantified by qRT-PCR 36 hours post transfection. Results represent the mean ± SE of three independent experiments (****p<0.0001 by 2-tailed t test). (b) Using a Flag-specific antibody or a negative control antibody (NC), NHEKs were immunostained for the Flag-tagged ST18 construct (red signal) in addition to DAPI (blue signal). (scale bar=20um).

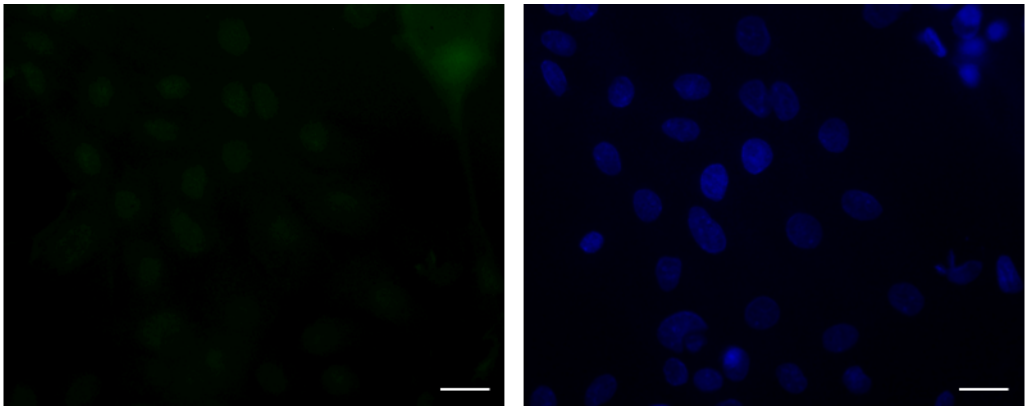

Supplementary Figure 5. Negative control for immunofluorescence staining.

DyLight 488 goat polyclonal anti rabbit secondary antibody (Invitrogen, Carlsbad, CA, USA, 35553) immunofluorescence staining in NHEKs which were fixed and immune-stained without the primary antibody as negative controls (blue staining, DAPI, right panel). (scale bar=20um)

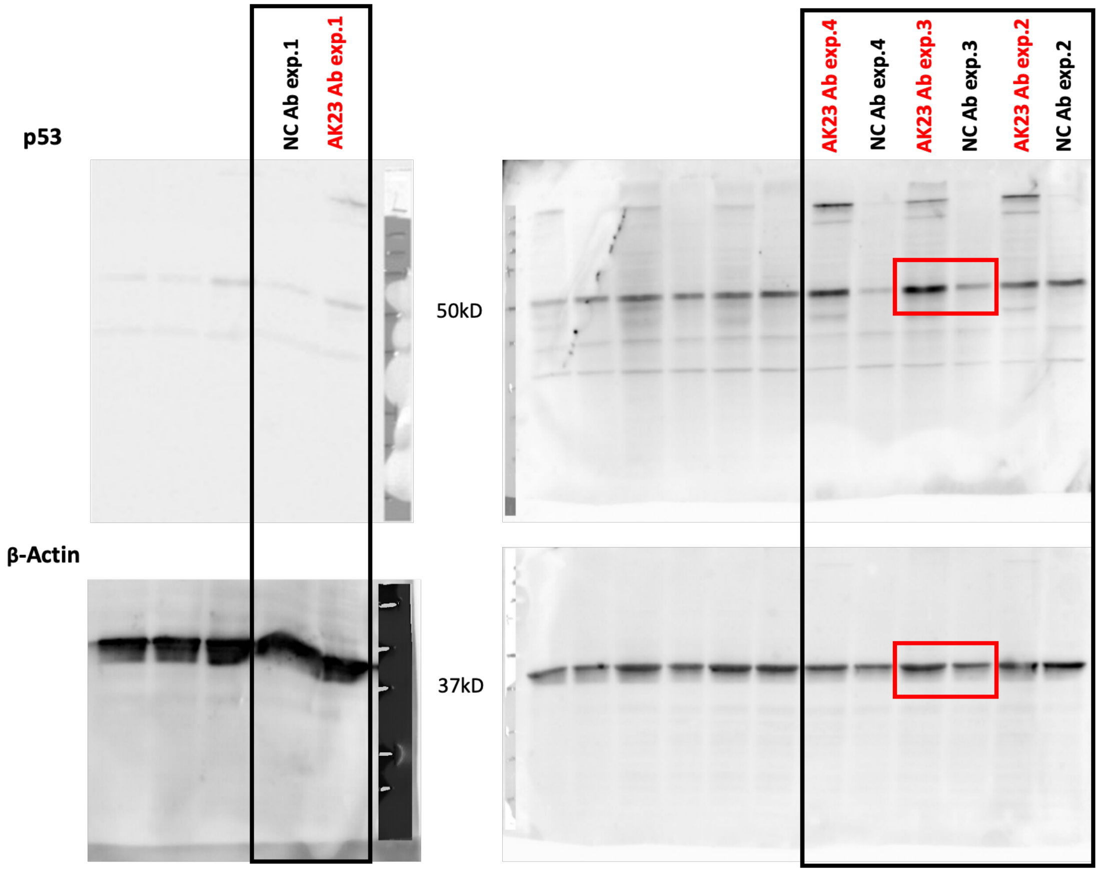

Supplementary Figure 6. Western blot full length membranes.

Full length membranes of the Western blots discussed in Fig.2b are provided; the relevant data are marked. A crop of experiment 3 (red boxes) is presented in Fig.2b.

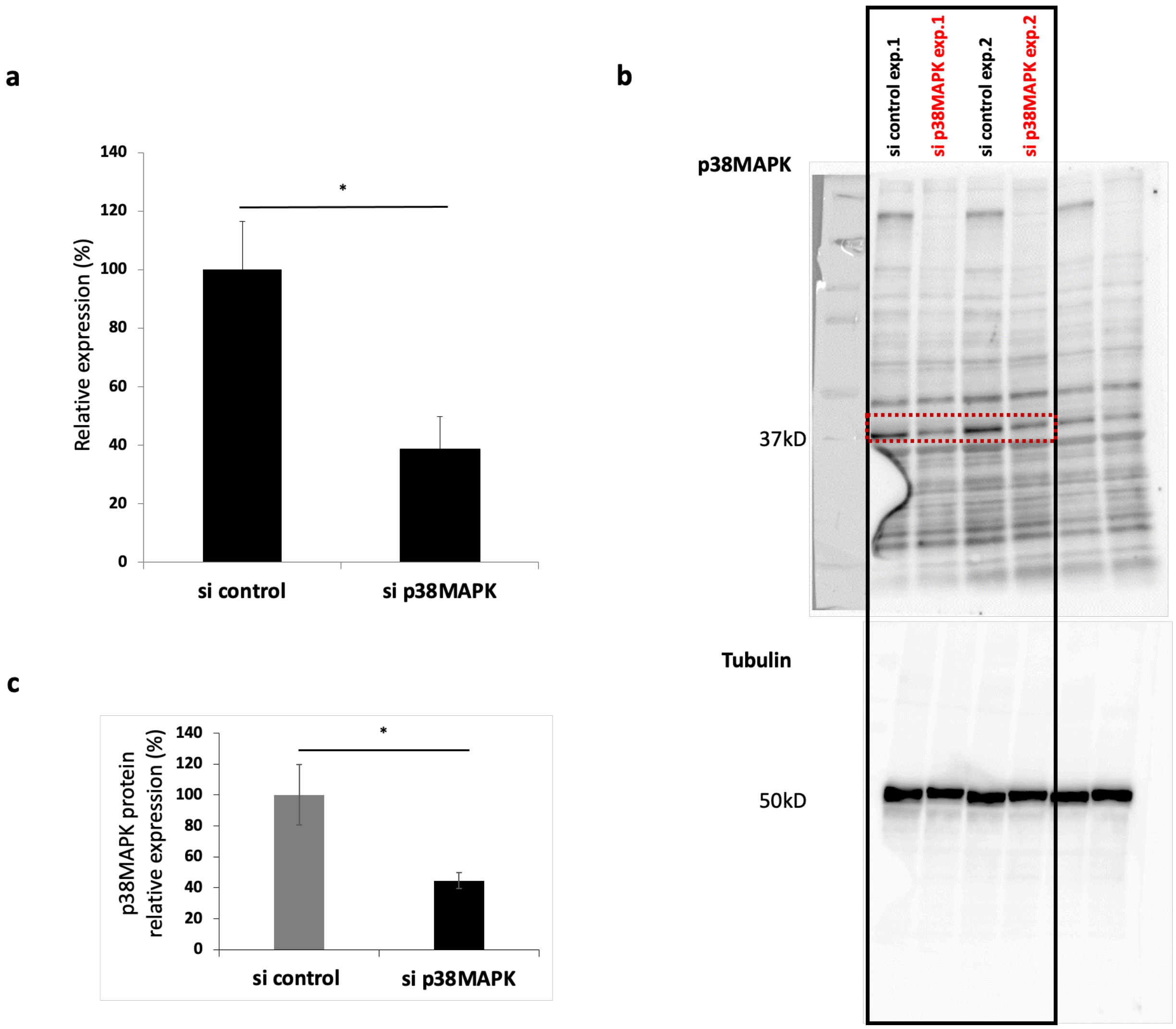

Supplementary Figure 7. p38MAPK silencing validation.

NHEKs were transfected as described in Materials and Methods with p38MAPK-specific siRNA (si p38MAPK) or control siRNA (si Control). (a) RNA levels were quantified by qRT-PCR 48 hours post transfection. Results represent the mean ± SE of four independent experiments (*p<0.05 by 2-tailed t test). (b) p38MAPK protein expression in NHEKs was assessed using immunoblotting with anti-p38MAPK antibody (red box). Tubulin served as a loading control. (c) Protein levels were quantified and data was normalized to levels observed in control siRNA-treated cells. Results represent the mean ± SE of two independent experiments (*p<0.05 by 2-tailed t test).

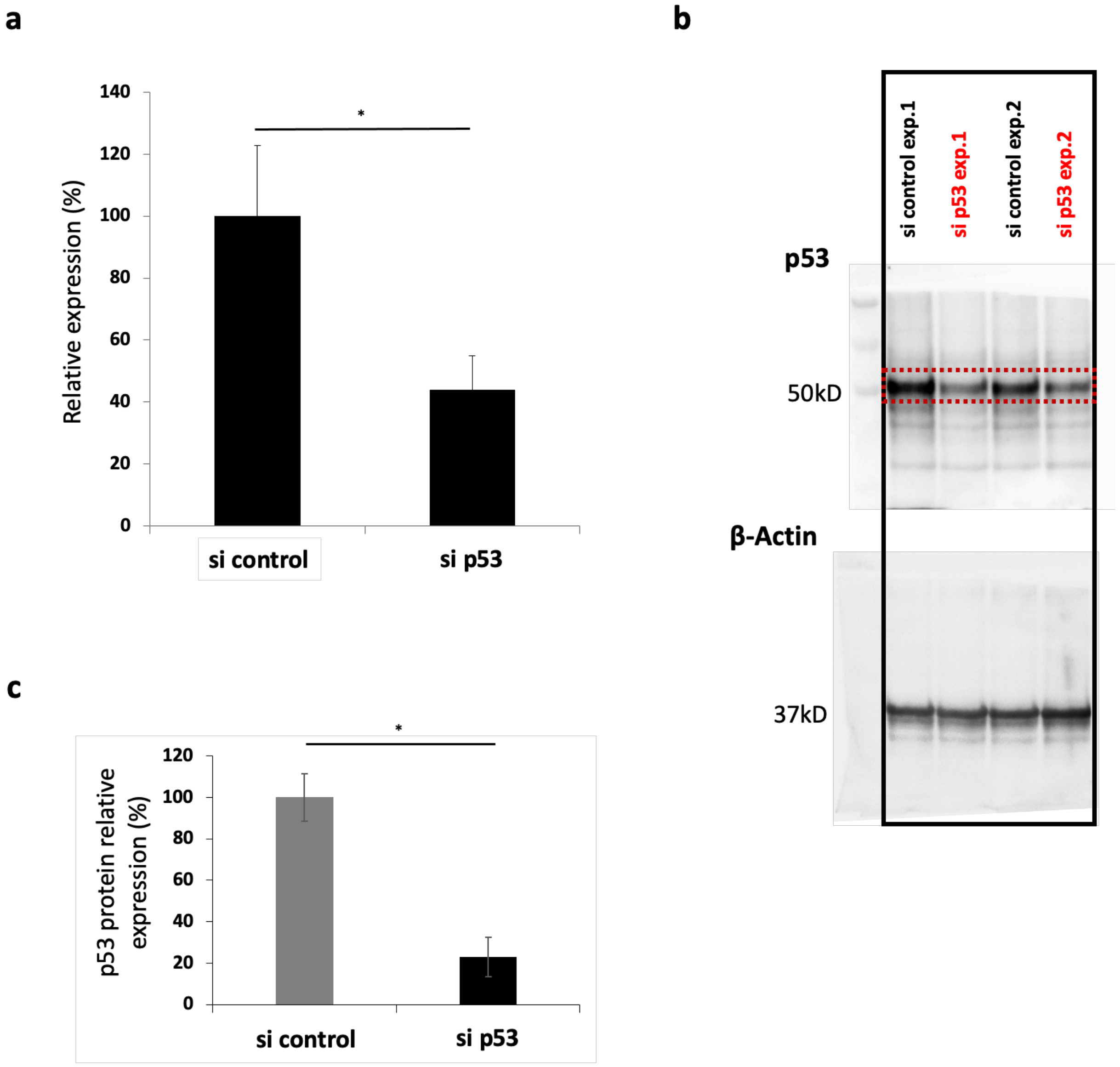

Supplementary Figure. 8. TP53 silencing validation.

NHEKs were transfected as described in Materials and Methods with TP53-specific siRNA (si p53) or control siRNA (si Control). (a) RNA levels were quantified by qRT-PCR 48 hours post transfection. Results represent the mean ± SE of three independent experiments (*p<0.05 by 2-tailed t test). (b) p53 protein expression in NHEKs was assessed using immunoblotting with anti-p53 antibody (red box). β-actin served as a loading control. (c) Protein levels were quantified and data was normalized to levels observed in control siRNA-treated cells. Results represent the mean ± SE of two independent experiments (*p<0.05 by 2-tailed t test).
